# Determinants of quality of life in individuals with chronic low back pain: a systematic review

**DOI:** 10.1080/21642850.2021.2022487

**Published:** 2022-01-05

**Authors:** Aleena Agnus Tom, Eslavath Rajkumar, Romate John, Allen Joshua George

**Affiliations:** Department of Psychology, Central University of Karnataka, Kalaburagi, India

**Keywords:** Quality of life, QOL, chronic low back pain, CLBP, determinants

## Abstract

**Objective:**

Chronic low back pain (CLBP) is a prominent medical condition that can affect an individual at some point in their life time which could lead to poor quality of life (QOL). Low back pain has affected approximately 577 million individuals globally by 2017. The aim of the current systematic review is to synthesise the existing evidence on the factors influencing the QOL in individuals with CLBP and to identify strategies to improve their QOL.

**Method:**

PubMed, ScienceDirect, PsychNet and Google Scholar were used to extract studies reporting quantitative relationships between QOL and its possible determinants in individuals having CLBP and the intervention strategies to improve QOL.

**Results:**

10,851 studies were initially identified and twenty-six studies which met the inclusion criteria were selected for the review. 21 studies reported relationship between QOL and potential determinants and five studies assessed the influence of interventions on QOL. Determinants were classified as kinesiophobia, fear avoidance belief, or pain belief; occupation-related factors; pain and disability; activity; personal factors including age, gender, employment status; and other psychological factors including anxiety, quality of sleep, and health locus of control. Intervention strategies including MBSR, Pilates method and Back School Programme improved QOL in individuals with CLBP.

**Conclusion:**

Psychosocial factors as well as the physical status of the individual contributed to the QOL in individuals having CLBP.

## Introduction

Low back pain is a prominent health condition that affects an individual at some point of their life (Hong, Kim, Shin, & Huh, 2014) and it is a major condition leading to disability affecting the work performance and the overall well-being of the individual (Ehrlich George, 2003; Manchikanti, Singh, Falco, Benyamin, & Hirsch, 2014). It is also a crucial health condition prevalent in most of the developing as well as developed countries leading to their economic and social decline (Montazeri & Mousavi, 2010). Low back pain is defined ‘as pain, muscle tension or stiffness localised below the costal margin and above the inferior gluteal folds with or without leg pain’ (Chou, 2010). LBP could be acute, sub-acute or chronic based on the severity (Ehrlich George, 2003). Low back pain becomes chronic when it persists for a period of 12 weeks or more (Mostagi et al., 2015). It affects people in all ages starting from early teen ages (Leboeuf-Yde & Kyvik, 1998) to adulthood (Calvo-Muñoz, Gómez-Conesa, & Sánchez-Meca, 2013) and is a very frequent reason for medical consultations.

Globally, LBP is identified to be the greatest contributor to global disability which was estimated by the 2010 Global Burden of Disease Study. Studies reveal that LBP takes a spot in the top 10 diseases that is accountable for the highest number of DALYs (Disability adjusted life years) globally (Hoy et al., 2014). According to the Global Burden of Diseases Study 2017, the all age and sexes combined number of prevalent cases of low back pain was 577 million (James et al., 2018) and females had a higher prevalence than males (Hoy et al., 2012). The LBP prevalence was 22.3 percent in child/adolescent population (Moncer et al., 2016), and it was around 15 per cent in adults and 27 per cent in the older adults (Manchikanti et al., 2014).

Quality of life is a very broad and multifaceted concept (Chaturvedi & Muliyala, 2016). It is considered to be synonymous with health status, physical functioning, perceived health status, perception of health, subjective health, well-being and functional disability (Passier, Visser-Meily, Rinkel, Lindeman, & Post, 2013). WHO defines the quality of life as ‘an individual's perception of their position in life in the context of the culture and value systems in which they live, and in relation to their goals, expectations, standards and concerns’ (WHO, 1997). Existing literature mention three approaches in conceptualising QOL, ‘1) equating quality of life with health, 2) equating it with well-being, and 3) by treating quality of life as superordinate construct’ (Post, de Witte, & Schrijvers, 1999). Health-related quality of life (HRQOL) is considered to be a part of the overall quality of life which includes the aspects of QOL that are related to the health of the individual.

CLBP is one of the most prevalent chronic pain disorders associated with a high burden on individuals and the society (Grabovac & Dorner, 2019), that can have a huge influence on the individual's QOL. There is an inevitable concern among the CLBP patients regarding the maintenance of QOL. Some cross-sectional studies have pointed out that low back pain has an inverse relationship with HRQOL (Ayranci, Tozun, & Unsal, 2010; Pedisic, Pranic, & Jurakic, 2013; Suka & Yoshida, 2008; Yamada et al., 2014). Literature states that CLBP has a strong association with high intensity of pain and disability, less prognosis rate, poorer HRQOL and significant physical limitations (Mutubuki et al., 2020). It was also revealed that the individuals affected with CLBP report a low QOL that is comparable to people diagnosed with life-threatening diseases (Fredheim et al., 2008). Moreover, research suggests that, even chronic low back pain patients who have lower levels of pain and disability also reported less quality of life (Urquhart, Shortreed, Davis, Cicuttini, & Bell, 2009). Studies have also focused on identifying the factors influencing QOL of CLBP patients.

The individuals with CLBP, experience persistent pain and disability at some stage in their life and report serious impairment in their QOL. Hence an exploration of the factors that lead to poorer quality of life in this diagnostic group will contribute to the existing scientific literature. Moreover, in order to design and test future interventions to improve the quality of life among individuals with CLBP, it is imperative to understand the major factors contributing to their quality of life. To the researchers’ knowledge, no systematic review on determinants of QOL in CLBP patients has been conducted to date. Therefore, the current study aims to synthesise the existing empirical evidence on the determinants of QOL in individuals having CLBP and to identify intervention strategies to improve their QOL most effectively.

## Methods

### Search strategy

A systematic search for the literature published from 2010 to 21st October 2020 was performed in PubMed, ScienceDirect and PsycNet. Manual searches for studies were conducted in Google Scholar to identify additional studies which are relevant to the study area. The search terms used were, Chronic Low back pain, Low back pain, CLBP, Specific chronic low back pain, non-specific chronic low back pain in combination with the quality of life, health-related quality of life (HRQOL), mental quality of life, QOL, HRQOL. The search terms were combined differently for the databases. The search terms were kept as broad as possible to ensure the inclusion of a maximum number of relevant articles. For example; Science direct: (Quality of life) AND (Chronic Low back pain). A similar search strategy was executed for identifying the determinants as well as intervention strategies.

### Study selection and eligibility criteria

The screening of the study titles and abstracts were done initially. After the initial screening, the full-text articles were assessed for selection. The criteria for inclusion were, (1) the study population exclusively concerned with chronic low back pain, (2) study that reported the relationship between possible determinants and QOL, (3) studies that reported interventions to improve quality of life and (4) articles published in the English language. The exclusion criteria include (1) use of a non-standardised measure of QOL, (2) pharmacological intervention studies, (3) articles published before 2010, and (4) Qualitative studies. Due to the higher number of quality of life research articles published every year, the article search was restricted to articles published in the previous 10 years and hence studies from 2010 were included in the review.

The studies were extracted from quality assured databases such as ScienceDirect, PubMed and PsycNet which ensured access to high-quality peer-reviewed articles. Besides, for assessing the quality of the intervention studies, Revised Cochrane risk-of-bias tool for randomised trials (RoB 2) was used which ensured a low risk of bias for the studies included. RoB 2 tool was structured into five domains including; (1) bias arising from randomisation process, (2) bias due to deviation from intended intervention, (3) bias due to missing outcome data, (4) bias in the measurement of the outcome, and (5) bias in the selection of the reported result. The risk of bias assessment done for the five intervention studies is given in Table 1.

**Table 1.** Risk of bias assessment (RoB 2).

	Randomisation	Effect of assignment to intervention	Effect of adhering to intervention	Risk of bias due to missing outcome data	Risk of bias in measurement of outcome	Risk of bias in selection of the reported results
Banth and Ardebil	Low	Low	Low	Low	Low	Low
Kofotolis et al.	Low	Low	Low	Low	Low	Low
Masumian et al.	Low	Low	Low	Low	Low	Low
Morone et al.	Low	Low	Low	Low	Low	Low
Natour et al.	Low	Low	Low	Low	Low	Low

### Data collection and analysis

As the preliminary step, the obtained data were merged and de-duplicated. After the duplicates were removed, titles and abstracts were examined to remove the articles irrelevant to the current study. Thirty per cent of the same were randomly cross-verified by the co-authors. The full-text documents were retrieved and were examined in detail in compliance to the inclusion criteria.

Information related to the research design, sample size, male-female ratio, duration of low back pain symptoms reported, the measurement tools used, and the assessed determinants of quality of life were collected and tabulated. All reported associations between factors and quality of life were taken into account. All the interventions other than the pharmacological interventions were taken into consideration. Correlation coefficients were the statistic that was mostly reported in the studies selected. Meta-analysis was not conducted, due to the diversity of the primary studies.

## Results

### 1. Search results

**Figure 1.**
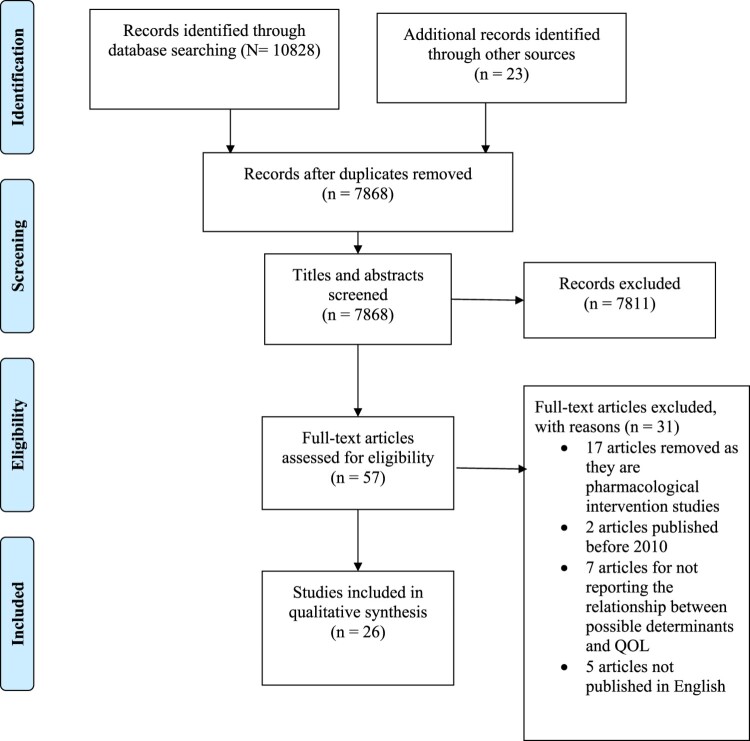
PRISMA flow diagram.

Literature search was conducted in Science Direct (9848), PubMed (976) and PsycNet (4). The PRISMA 2009 flow diagram for the systematic search conducted is shown in Figure 1. A manual search for literature was done in Google Scholar and 23 articles were identified. After the removal of duplicates, 7868 abstracts were checked for inclusion. 7811 studies were eliminated based on the screening of titles and abstracts. 57 full-text articles were considered for eligibility. 17 articles were eliminated as they were pharmacological intervention strategies, two articles were excluded as they were published before 2010, seven studies were excluded for not reporting association between possible determinants and QOL, and five articles were excluded as they were not in English. After the screening of full texts, 26 articles which satisfied the inclusion criteria were selected. 21 selected studies discussed possible determinants of QOL and 5 studies discussed intervention strategies to improve QOL in individuals having CLBP.

### 2. Study characteristics

Study characteristics are summarised in Table 2. The sample size of the 26 articles ranged from 18 participants to 6316 and the total number of participants were 12,568. 18 out of the 26 studies have followed cross-sectional research design, 5 used experimental design and 2 were cohort studies. The studies were conducted in 16 different countries including Turkey (n = 6), Brazil (n = 3), Japan (n = 2), Netherlands (n = 2), France (n = 1), Germany (n = 2), Iran (n = 1), Italy (n = 1), China (n = 1), Cameroon (n = 1), South Korea (n = 1), Slovenia (n = 1), Indonesia (n = 1), Poland (n = 1), India (n = 1) and Greece (n = 1). A total of seven different Quality of life measurements were used. 36-item Short Form Health Survey was used as a measure of the quality of life in 12 studies. SF-36 is a 36 item questionnaire which measures eight domains of quality of life including physical functioning, physical role limitation, bodily pain, social functioning, general mental health, emotional role limitations, vitality or energy, general health perceptions. RAND-36 was another measure used which is similar to SF-36 except for scoring. WHOQOL as well as WHOQOL-BREF were also used as a measure of QOL in some studies. WHOQOL consisted of six domains which had different facets. The domains included physical, psychological, level of independence, social relationships, environment, and spirituality/religion/personal beliefs. WHOQOL-BREF had 4 major domains including; physical health, psychological, social relationship and environment which includes financial resources, home environment, freedom and security etc. as the facets. SF-12 was also used as a measure of QOL in some studies.

**Table 2.** Study characteristics of the articles reviewed.

Authors, year, nationality	Study design	Participants (n)	Sample characteristics	Assessment tool	Determinants
Altuğ et al., 2016, Turkey	Cross-sectional study	112 patients	73 females & 39 males, CLBP > 3 months	VAS, IPAQ, TKS, ODI, SF-36	Kinesophobia (Significant negative relation with sub-parameters of SF-36)
Alaca et al., 2020, Turkey	Cross-sectional study	89 patients	47 males & 42 females, CLBP > 6 months	VAS, PBQ, TKS, ODI, SF-36	Kinesiophobia inverse correlation with mental health sub-dimension. Organic pain beliefs correlated with all sub-dimensions except physical limitation and role limitation. Psychological pain beliefs negative correlation with vitality and social function sub-dimension & positive correlation with mental health sub-dimension
Tsuji et al., 2018, Japan	Cross-sectional study	239	84 female & 155 male, CLBP> 3 months, employed	work productivity and activity impairment questionnaire, PHQ, SF-36	High presenteeism leading to impairments in HRQOL (*P* < 0.001)
Guclu et al., 2012, Turkey	Cross-sectional study	105	69 female, 36 male, CLBP for at least 3 months,	VAS, BAI, BDI, FABQ, SF-36, Rolland Morris Disability questionnaire	Pain severity, Fear avoidance (overall, physical and work), Anxiety – negative significant correlation with physical function, role function physical/emotional sub-dimensions of SF-36.
Comachio et al., 2018, Brazil	Cross-sectional study	132	40 males & 92 females, CLBP> 3 months	TSK, Rolland Morris questionnaire, McGill pain questionnaire, SF36	Kinesophobia correlated with physical and emotional role limitation.
Du et al., 2018, China	Cross-sectional study	221	127 females & 94 male, Pain for more than 3 months,	SES-6, FAB questionnaire, coping styles questionnaire, SF-36	Self-efficacy, Fear avoidance belief, active and passive coping predicting physical component summary of QOL. SE, passive coping and FAB predicting mental component summary of QOL.
Aminde et al., 2020, Cameroon	Cross-sectional study	136	Female: male = 1.8:1, CLBP > 3 months	VAS, WHOQOL-BREF, RMDQ	Higher perceived pain intensity, work absence, high score for disability, higher reported pain intensity, current smoking, duration of pain episode, education, income.
Stefane et al., 2013, Brazil	Cross-sectional study	97	67 female & 30 male,	RMDQ, WHOQOL Brief, 11 point scale of pain intensity,	Pain intensity weak negative correlation with physical domain of QOL. Disability has an inverse correlation to the physical domain and a moderate negative correlation with psychological domain of QOL.
Fujii et al., 2018, Japan	Cross-sectional study	3100	1483 female & 1617 male, CLBP> 3 months,	SSS-8, PHQ-2, EQ-5D-3L	Somatic symptoms scores were negatively correlated with QOL.
Huijnen et al., 2011, Netherlands	Cohort study	116		HSQ; 12, BDI, Baecke Physical Activity Questionnaire, RDQ, RAND-36,	Avoider activity related style has increased mental QOL.
Jung et al., 2018, South Korea	Cross-sectional study	108		VAS, ODI, SF-36, BDI,	Depression, sex, knee extension with dorsiflexion, and age
Mutubuki et al., 2020	Cohort study	6316	Female percentage = 66%	Numeric pain rating scale, ODI, EQ-5D-3L, cost questionnaire.	Pain intensity (negative relation), Disability (negative relation).
Sengul et al., 2010, Turkey	Cross-sectional study	113	41male& 72 female, CLBP> 3 months	VAS, ODI, MHLC, WHOQOL	Health locus of control. Internal health locus of control and chance locus of control.
Ketiš, 2011, Slovenia	Cross-sectional study	187	45.5% of men, CLBP lasted more than 3 months.	VAS, EQ-5D, ODI,Duke-AD	Higher level of chronic pain associated with lower QOL, presence of anxiety and depression,
Schaller et al., 2015, Germany	Cross-sectional study	412	96 males &316 females.	EQ-5D, GPAQ,	Moderate and rigorous workplace physical activity (negative association), Leisure time, pain intensity
Semeru & Halim, 2019, Indonesia	Cross-sectional study	52	43 females, 9 males	PDM,CPAQ-R, PCS, NEO FFI	Catastrophising positively related to QOL dimensions, Acceptance
Sezgin et al., 2015, Turkey	Cross-sectional study	200		SF-MPQ, FRI,SF 36, PSQI	Sleep quality (negative correlation with physical component summary scores)
Thomas et al., 2010, France	Cross-sectional study	50	Female % = 30%. CLBP for at least 3 months.	RMDQ, DPQ, FABQ, TSK, PCS, HAD.	Approximately 73% reporting impaired QOL. Psychosocial factors predict disability and QOL with catastrophising or kinesiophobia
Uchmanowicz et al., 2019, Poland	Cross-sectional study	100	chronic back pain lasting longer than 3 months	VAS, ESS, AIS, WHOQOL-BREF	Sex, age, place of residence, education, marital status, professional activity and duration of illness. Insomnia predicts QOL.
Ünal et al., 2019, Turkey	Cross-sectional study	114	86 females & 28 males, chronic back pain lasting longer than 3 months	IPQ-R, VAS, 6MWT, ODI, BDI, SF-36	Illness perception
Wettstein et al., 2019, Germany	Cross-sectional study	228	Female % = 71.5	MPI-D, PIS, SF-12, HADS-D	Age was associated with QOL related to mental health and well-being.

### 3. Determinants of quality of life

The possible factors determining the quality of life among patients with CLBP were assessed in 21 of the 26 studies considered for the review. All the determinants identified as a result of the review were listed by the first author initially and was later clubbed into categories depending on their relative similarities.

#### 3.1. Kinesiophobia, pain belief and fear avoidance belief

Kinesiophobia, pain belief and fear avoidance belief (FAB) were assessed in six out of the 21 studies summarised in Table 2 (Alaca, Kaba, & Atalay, 2020; Altuğ et al., 2016; Comachio, Magalhães, Campos Carvalho E Silva, & Marques, 2018; Du et al., 2018; Guclu, Guclu, Ozaner, Senormanci, & Konkan, 2012; Thomas et al., 2010).

##### 3.1.1. Kinesiophobia

Kinesiophobia refers to ‘avoidance behaviour due to fear’ or ‘an excessive, irrational, and debilitating fear of physical movement and activity resulting from a feeling of vulnerability to painful injury or re-injury’ (Demirbüken et al., 2016). Kinesiophobia was measured using Tampa Scale for Kinesiophobia (TKS) which was a 17 item self-report questionnaire developed by Miller, Kori and Todd in 1991 for assessing the fear of movement of the individuals (Comachio et al., 2018).

Four studies reported a negative correlation between kinesiophobia and sub-dimensions of quality of life. Highly significant inverse correlation was reported between kinesiophobia and sub-dimensions of SF-36 including general health, physical function and social function roles restricted by physical function and bodily pain (Altuğ et al., 2016) while a negative correlation was found with the mental health sub-dimension of SF-36 (Alaca et al., 2020). Kinesiophobia was also correlated with physical role limitation and emotional role limitation dimensions of quality of life index (Comachio et al., 2018).

##### 3.1.2. Fear avoidance belief

‘Fear avoidance is a belief that any movement or activity that may provoke pain should be avoided due to the fear of causing pain or re-injury’ (Du et al., 2018). Fear Avoidance Belief was assessed using Fear Avoidance Belief questionnaire (FABQ). FABQ was developed by Waddel et al in 1993 based on the fear avoidance belief model and consists of 16 items and two sub-domains including work subscale and physical activity subscale (Du et al., 2018).

Fear avoidance belief overall and the sub-domains of fear avoidance including fear avoidance-physical and fear avoidance-work were negatively correlated with the domains of SF-36 including physical function, role limitation due to physical health problems and role limitation due to emotional problems (Guclu et al., 2012). Fear avoidance was also reported to be the predictor of physical health component summary of QOL and mental health summary of QOL assessed using SF-36 (Du et al., 2018).

##### 3.1.3. Pain belief

Pain belief refers to ‘patients’ beliefs about the cause of their pain and the anticipated effects of the treatment’ (Walsh & Radcliffe, 2002). Pain beliefs were assessed using Pain Belief Questionnaire (PBQ) which is a 20 item questionnaire developed by Edward et al to assess the beliefs about the sources and outcome of pain in chronic pain patients (Edwards, Pearce, Turner-Stokes, & Jones, 1992). The pain beliefs were assessed as organic beliefs and psychological beliefs (Alaca et al., 2020).

Organic pain belief was associated with all sub-dimensions of SF-36 including physical function, bodily pain, general health, vitality, emotional role limitation and mental health; while psychological pain beliefs were negatively correlated with vitality and social function sub-dimension and positively correlated with mental health sub-dimension of SF-36 (Alaca et al., 2020).

#### 3.2. Occupation-related factors

Three studies assessed the relationship between occupation-related factors and QOL in people having CLBP (Aminde et al., 2020; Schaller, Dejonghe, Haastert, & Froboese, 2015; Tsuji, Matsudaira, Sato, Vietri, & Jaffe, 2018). The questionnaires used were Work Productivity and Activity Impairment (WPAI) Questionnaire and Global Physical Activity Questionnaire (GPAQ). WPAI is a six-item questionnaire which measures four domains such as absenteeism, presenteeism, overall work productivity loss and activity impairment (Tsuji et al., 2018). GPAQ is a 16 item questionnaire which assesses three domains of physical activity including physical activity at work, transport activity and recreational activity (Schaller et al., 2015).

##### 3.2.1. Presenteeism

Presenteeism refers to attending work while being physically ill (Johns, 2010). People with high presenteeism reported a greater HRQOL burden (Tsuji et al., 2018).

##### 3.2.2. Absenteeism

Workplace absenteeism refers to an employees’ intentional or habitual absence from work (Cucchiella, Gastaldi, & Ranieri, 2014). Longer days of work absence were also negatively correlated with health-related quality of life (Aminde et al., 2020).

##### 3.2.3. Workplace physical activity

Moderate and rigorous workplace physical activity had an inverse correlation with QOL (Schaller et al., 2015).

#### 3.3. Pain and disability

Pain intensity and severity and disability were also prominent determinants of QOL. Five studies assessed the relationship between QOL and these variables among people with CLBP (Aminde et al., 2020; Guclu et al., 2012; Ketiš, 2011; Mutubuki et al., 2020; Stefane, Santos, Marinovic, & Hortense, 2013). Rolland Morris Disability Questionnaire (RMDQ) measured disability, and pain intensity and severity using Visual Analogue Scale (VAS). RMDQ is a 24 item questionnaire developed by Rolland and Morris in 1983 for assessing the functional impairment in individuals (Roland & Morris, 1983). VAS is a validated 10-point rating scale which represents a continuum between ‘no pain’ and ‘worst pain’ (Price, McGrath, Rafii, & Buckingham, 1983).

Pain severity had a significant inverse correlation with physical function, physical role functioning and emotional role functioning sub-dimensions of SF-36 (Guclu et al., 2012). Higher perceived pain severity score, higher reported pain intensity, and high disability scores had an inverse correlation with HRQOL (Aminde et al., 2020; Mutubuki et al., 2020). Pain intensity had a correlation with the physical domain of QOL (Stefane et al., 2013). Greater chronic pain was correlated to lower QOL (Ketiš, 2011).

#### 3.4. Physical activity

Three studies found activity as a determining factor in the QOL of individuals with CLBP (Huijnen et al., 2011; Jung et al., 2018; Schaller et al., 2015). Global Physical Activity Questionnaire (GPAQ) was used as a measure of physical activity as well as Baecke physical activity questionnaire (BPAQ). BPAQ was used as a measure of habitual physical activities in daily life and the instrument gives three indices of habitual physical activity including occupational activity, sports activity and leisure activity index (Huijnen et al., 2011).

Patients characterised by avoidant activity style with an increment in the daily uptime showed improvement in mental health-related QOL (Huijnen et al., 2011). Knee extension with dorsiflexion might interfere with activities of daily living and affecting the quality of life as a result (Jung et al., 2018). Moderate and rigorous workplace physical activity had an inverse relationship with quality of life and patients achieving WHO recommendations of leisure time physical activities (≥600 MET-min/week) reported better HRQOL than those reporting no leisure time physical activities (Schaller et al., 2015).

#### 3.5. Personal factors

##### 3.5.1. Demographic characteristics and health status

Age, gender, employment status and health status including duration of illness, smoking habits were identified to be related with QOL.

###### 3.5.1a. Gender

Two studies revealed that females reported reduced quality of life than males (Jung et al., 2018; Uchmanowicz, Kołtuniuk, Stępień, Uchmanowicz, & Rosińczuk, 2019).

###### 3.5.1b. Age

Age was identified to be a significant predictor of mental health dimension of health-related quality of life which was measured by SF-12 (Jung et al., 2018; Wettstein, Eich, Bieber, & Tesarz, 2019) while another study revealed that age does not affect any domains of QOL assessed by WHOQOL-BREF (Uchmanowicz et al., 2019).

###### 3.5.1c. Employment status

QOL in the social domain were higher for those employed compared to the unemployed (Uchmanowicz et al., 2019).

###### 3.5.1d. Health status

Duration of illness and pain episode was associated with poorer QOL (Aminde et al., 2020; Uchmanowicz et al., 2019). Current smoking had an association with HRQOL (Aminde et al., 2020).

##### 3.5.2. Other psychological characteristics

###### 3.5.2a. Anxiety

Anxiety was assessed using the Beck anxiety inventory which is a 21 item instrument for assessing the level of anxiety (Guclu et al., 2012). Anxiety was found to have a significant inverse correlation with physical function, role function physical/emotional sub-dimensions of SF-36 (Guclu et al., 2012). The presence of anxiety and depressive symptoms were also associated with poor quality of life (Ketiš, 2011).

###### 3.5.2b. Self-efficacy

Self-efficacy was assessed using Self-efficacy for managing chronic pain on a 6-item scale (SES-6). SES-6 measures two dimensions including self-efficacy for managing symptoms and self-efficacy to manage disease in general (Du et al., 2018). Self-efficacy was a determinant of the physical component and mental component summary of QOL (Du et al., 2018).

###### 3.5.2c. Coping styles

The measure used for assessing the coping styles was simplified coping styles questionnaire (SCSQ). The scale consists of 20 items with two dimensions including passive coping styles and active coping styles (Du et al., 2018). Active and passive coping styles were determinants of physical component and mental component summary of QOL (Du et al., 2018).

###### 3.5.2d. Locus of control

Locus of control was measured using the Multidimensional Health Locus of Control (MHLC) scale. Patients with chance health locus of control (CHLC) had a negative impact on the quality of life (Sengul, Kara, & Arda, 2010).

###### 3.5.2e. Catastrophising

Catastrophising was assessed using pain catastrophising scale (PCS). PCS is a 13-item self-report questionnaire consisting of three domains including rumination, helplessness and magnification (Semeru & Halim, 2019). Catastrophising had a positive association with quality of life dimensions including anger and frustration, fear and uncertainty which were measured using Pain and Discomfort module (PDM) (Semeru & Halim, 2019).

###### 3.5.2f. Sleep quality

Sleep quality measure used was Pittsburgh sleep quality index (PSQI). It is a 19-item self-report index having seen sub-scales evaluating sleep quality, sleep latency, sleep duration, habitual sleep efficiency, sleep disturbances, use of sleeping medications and day-time disturbances (Sezgin et al., 2015). Sleep quality was negatively correlated with a physical component summary of the quality of life (Sezgin et al., 2015; Uchmanowicz et al., 2019).

###### 3.5.2g. Illness perception

Illness perception was assessed using Illness Perception Questionnaire-Revised (IPQ-R). Positive illness perception was related to improved QOL (Ünal et al., 2019).

### 4. Intervention strategies

The study characteristics of the intervention studies are summarised in Table 3. Five intervention studies were identified as part of the review.

**Table 3.** Details of the intervention studies reviewed.

Authors, year, nationality	Study design	Participants (n)	Sample characteristics	Assessment tool	Interventions conducted
Banth & Ardebil, 2015, India	Pre-post quasi time series experimental design	88	Age 30–45 years females	McGill Pain questionnaire, Quality of life (SF-12),	MBSR has a significant impact on physical and mental QOL.
Kofotolis et al., 2016, Greece	Experimental design	101	Women aged 25–65 years,	SF 36	Patients given with Pilates exercises reported greater improvements in self-reported functional disability and health-related QOL.
Masumian et al., 2018, Iran	Pre-post-test experimental design with control group	18	Age 18–60 years	Multidimensional pain inventory, Five facet mindfulness questionnaire, FABQ	MBSR therapy is found to be effective in reducing fear avoidance belief
Morone et al., 2011, Italy	Randomised controlled trial	74		SF36, VAS, ODI,	Back school programme has produced significant improvement in several dimensions of SF36
Natour et al., 2015, Brazil	Randomised controlled trial	60		VAS, Rolland Morris questionnaire, SF36	Pilates method was found to be effective in improving dimensions of QOL

#### 4.1. Mindfulness-based stress reduction (MBSR)

Two studies assessed the effect of mindfulness-based stress reduction (MBSR) intervention on improving QOL of individuals who have CLBP. MBSR is a mindfulness-based eight-week group programme for individuals having a wide range of stress-related conditions (Baer, 2010). MBSR had a significant impact on physical and mental QOL (Banth & Ardebil, 2015) and was found to be effective in reducing fear avoidance belief (Masumian, Shairi, Ghahari, Rajabi, & Mazloumi, 2018).

#### 4.2. Pilate exercise method

Two studies assessed the effect of the Pilate exercise method on the QOL of individuals with CLBP. ‘Pilates method is a unique system of techniques including stretching and strengthening exercises developed to achieve better functioning of the body based on the strengthening of the lower trunk region’ (Natour, Cazotti, Ribeiro, Baptista, & Jones, 2015). Individuals administered with Pilate exercise intervention showed reduction in self-reported functional disability and improvement in HRQOL (Kofotolis, Kellis, Vlachopoulos, Gouitas, & Theodorakis, 2016; Natour et al., 2015).

#### 4.3. Back school programme

The Back school programme intervention was studied in one article. Back school is an exercise programme which is mostly used for the treatment of CLBP (Morone et al., 2011). Physical and mental composite score of QOL improved CLBP patients when they were administered with back school programme intervention, reported in one of the studies (Morone et al., 2011).

## Discussion

Low back pain is one of the prominent health condition affecting the quality of life of the individuals. It affects various domains of daily life from basic self-care activities to advance and complex social interactions, work and leisure activities and eventually leading to poor quality of life (Montazeri & Mousavi, 2010). The present systematic review was aimed to identify the determinants of QOL in patients with CLBP and the intervention techniques to improve their QOL. Various potential determinants of QOL were identified as a result of the review.

Considering the fear related to movement and associated factors, kinesiophobia, fear avoidance belief, and pain beliefs were identified to be significant predictors of QOL in individuals with CLBP. Studies revealed that kinesiophobia has a negative influence on QOL and is negatively associated with the dimensions of QOL. Excessive fear restricts them from creating further movements and restricting the activities of daily living (Alaca et al., 2020) and majorly contributing to deprivation in areas related to the physical aspects of QOL including physical role limitation, and was also negatively influencing the mental health aspect of QOL emerging from the excessive and irrational fear. The fear of movement restricts physical activities in individuals due to the vulnerability of causing severe pain and is affecting their quality of life negatively (Altuğ et al., 2016). Another comparative study revealed that individuals with low back pain developed severe kinesiophobia, regardless of the severity of the pain, resulting in lower physical activity levels and kinesiophobia had an adverse effect on their quality of life (Uluğ, Yakut, Alemdaroğlu, & Yılmaz, 2016). Longitudinal studies also revealed that high level of kinesiophobia predicts negative changes in quality of life and positive changes in disability and pain (Luque-Suarez, Martinez-Calderon, & Falla, 2019). Kinesiophobia is closely related to the concept of fear avoidance belief. Kinesiophobia refers to the fear of movement or re-injury (Vincent et al., 2013), while fear avoidance belief focuses specifically on beliefs about how physical activity and work affects the pain (Waddell, Newton, Henderson, Somerville, & Main, 1993). Studies revealed fear avoidance to be a predictor of physical health and mental health summary of the quality of life and are found to be negatively influencing both physical functioning and role functioning (Guclu et al., 2012). Fear avoidance is identified to be a prognostic factor for poor outcomes in low back pain patients and considered as a maintaining factor (Wertli et al., 2014) which is directing to poor QOL in people having CLBP. The individual’s beliefs related to the causes of the pain and the outcomes of the pain were identified to have a significant impact on the quality of life of the individual. Studies revealed that kinesiophobia and fear avoidance beliefs are factors that play a major role in the development of low back pain and its transition towards chronicity (Macías-Toronjo, Rojas-Ocaña, Sánchez-Ramos, & García-Navarro, 2020). Fear is the basic reaction to the identifiable threat (Rachman & Hodgson, 1974) and the associated escape behaviours such as activity avoidance become maladaptive in the long term. The fear and the associated beliefs to carry out activities in the context of chronic musculoskeletal pain causes a negative vicious cycle where the individuals experience greater pain, disability and emotional distress and thereby leading to poor quality of life (Luque-Suarez et al., 2019).

Pain-related factors are highly contributing to the quality of life among individuals with CLBP. Pain severity is negatively influencing physical function and role functioning. Greater pain severity and greater disability are contributing to poor quality of life outcomes (Aminde et al., 2020). Pain severity affects the daily living as well as the quality of life. Chronic pain leads to disability and absence from work (Farahani & Assari, 2010). Studies revealed that there is a tendency for poorer quality of life, higher pain severity and prognostic risk in individuals suffering from CLBP (Ferrer-Pena, Calvo-Lobo, Aiguadé, & Fernández-Carnero, 2018). In another study, pain was found to be affecting all domains of QOL mostly affecting the physical and emotional functioning, and the extent of the effect was determined by extent, intensity, chronicity, and duration of the pain (Niv & Kreitler, 2001). The severity of the pain and the associated functional disability is leading to the experiencing of poorer quality of life in individuals having CLBP. The occupation-related factors also imply the similar facts. Presenteeism (Tsuji et al., 2018) as well as rigorous workplace activity (Schaller et al., 2015) was found to be significantly associated with poor QOL. An individual diagnosed with CLBP tends to have issues of performance in their workspace, that could possibly have an impact on their quality of life. Studies revealed that the existence of chronic pain in workers is a major economic burden (Phillips, 2009) and the need to continue to work despite the chronic pain is leading to lower productivity and poorer quality of life (Yamada, Matsudaira, Imano, Kitamura, & Iso, 2016).

Physical activity is also an important determinant of quality of life. Studies revealed that an avoidant activity style (Huijnen et al., 2011) and moderate and rigorous work place activity (Schaller et al., 2015) to be contributing to poor QOL. Studies revealed that activity avoidance does not promote healthy functioning in individuals with chronic pain, rather it leads to lower level of physical activity and greater physical disability (McCracken & Samuel, 2007) resulting in experiencing poor QOL. Studies also revealed that high activity along with an avoidant activity style leads to increased functional disability leading to poor QOL (McCracken & Samuel, 2007).

The current review is able to identify other personal factors contributing to the quality of life among individuals with CLBP. Sociodemographic factors influencing the quality of life are identified as part of the review including, gender, age, duration of illness, employability, and presence or absence of smoking. Studies reveal that mental health-related QOL was better among older individuals compared to younger patients (Wettstein et al., 2019). Other personal factors identified include psychological characteristics including the presence of symptoms related to depression and anxiety. Having symptoms of anxiety and depression predicted poor QOL. This could also be related to the presence of fear avoidance belief which was associated with depression as well as anxiety (de Moraes Vieira et al., 2014).

Other factors such as self-efficacy, coping styles and locus of control were also influencing the quality of life of CLBP patients (Du et al., 2018). Studies pointed out that individuals who have adopted passive coping strategies were found to have double the level of disability as compared to persons adopting active coping strategies (Blyth, March, Nicholas, & Cousins, 2005). Even though reported in one study, these factors suggested a significant relationship with QOL dimensions. Obeying the WHO leisure time physical activity recommendation was found to be contributing to a better quality of life (Schaller et al., 2015). Poor sleep quality predicted poor quality of life (Uchmanowicz et al., 2019). Studies had also pointed out that sleep problems including poor subjective sleep quality and short duration of sleep were predicting poor QOL (Lo & Lee, 2012). Another important determinant identified was locus of control. Patients having the chance locus of control were reported to have poor quality of life (Sengul et al., 2010). Those people having the chance locus of control tend to hold fatalistic beliefs and believes in fate regarding health and illness (Wallston, Strudler Wallston, & DeVellis, 1978), and studies revealed that people who believed in external factors such as chance and health professionals were more susceptible to disability (Mackenbach, Borsboom, Nusselder, Looman, & Schrijvers, 2001) and thereby having poor QOL (Kovacs et al., 2004). Catastrophising was also an important factor influencing the QOL in patients with CLBP. Studies showed that catastrophising caused patients to feel frustrated, develop anxiety and fear and thus leading to poor QOL (Severeijns et al., 2001).

These were the possible determinants of quality of life identified through the review. The psychological profile of the individuals having CLBP is one of the most important prognostic predictor. Studies revealed that patients’ cognitive behavioural profile predicts the prognosis and the extent of psychological distress within the individual indicating the QOL (Trocoli & Botelho, 2016). Studies revealed that bringing a change in the individual’s attitude towards pain can reduce the level of pain and its impact on the QOL (Mills, Nicolson, & Smith, 2019).

Five intervention studies were analysed during the review. MBSR was effective in improving the quality of life of CLBP patients. MBSR was identified to have a significant effect in reducing the fear avoidance belief (Masumian et al., 2018) which is one of the key determinants of QOL and to improve physical and mental quality of life (Banth & Ardebil, 2015). MBSR is capable of reducing the negative effect of symptoms of depression and anxiety as well as fatigue and pain understanding and thereby improving QOL (Diaz & Wolf., 2013). Several other studies have pointed out that MBSR has a significant effect on different psychological condition including reduction of psychological symptoms of distress, anxiety, depression and rumination (Bohlmeijer, Prenger, Taal, & Cuijpers, 2010; Carlson, Speca, Patel, & Goodey, 2003; Jain et al., 2007) which were the determinants of QOL and thereby improving the QOL (Bakhshani, Amirani, Amirifard, & Shahrakipoor, 2015).

Another intervention technique reviewed was the Pilates method. The review revealed that the Pilates method is contributing to improvement in quality of life and its various dimensions (Masumian et al., 2018). It also revealed that the Pilates method is effective for patients suffering from LBP (Natour et al., 2015). Studies revealed that Pilates emphasises on breathing control and muscle stretching which is assumed to have a positive psychosomatic impact on patients, thereby contributing to a better self-reported HRQOL (Masumian et al., 2018). In another study, it was reported that exercises such as Pilates which are trying to associate the mind and body, also influences the psychosocial, spiritual, emotional and behavioural characteristics of the individual and thereby reducing the psychological distress and the anxiety and depressive experiences which has a direct impact on the QOL (Cordeiro et al., 2020). Another intervention strategy identified was back school programme. Back school is an exercise programme which is mostly used for the treatment of CLBP (Morone et al., 2011). Studies revealed that Back School Programme is effective in improving the physical as well as the psychological status of the individual which were the determinants of QOL in individuals with CLBP (Tavafian, Jamshidi, & Montazeri, 2008).

These were the major intervention strategies identified which were effective in improving the QOL of people who were diagnosed to have CLBP. These intervention strategies were successful in dealing with some of the possible determinants of QOL such as FAB, anxiety, disability and pain in CLBP patients. But the intervention strategies identified were not sufficient to cover all the possible determinants of quality of life. One among these interventions alone will not cover all the factors that are influencing the quality of life. Hence, it will be more useful if an intervention which covers all or most of the determining factors of QOL for individuals with CLBP is introduced as part of future exploration in this area. As there were only a few intervention studies identified, any conclusions regarding their efficiency on improving the quality of life could not be made.

The current review also has some limitations. Firstly, heterogeneity in the sample studies in terms of research design, sample size and measurement instruments could have affected the results. Another limitation is that the literature search was limited to the English language and therefore the study might have overlooked similar studies published in other languages. Also, some studies included in the review had a low sample size and hence it can affect the conclusions drawn from the review. As the studies considered were conducted in different countries, the conclusions drawn could not be generalised while ignoring the cultural differences.

As part of the future exploration, a cross-cultural exploration of the differences in the determinants of quality of life in the context of CLBP could be made. A met analysis could be done to combine the results and thus to derive statistically driven conclusions regarding the determinants of QOL.

## Conclusion

The current systematic review is successful in identifying the possible determinants of QOL in individuals having CLBP. The current review provides a valuable contribution to the literature by summarising the results of the studies investigating the determinants of QOL in the context of CLBP. The psychological status of the individual was a significant contributor to the QOL of individuals with CLBP. The review identified various psychological determinants of QOL in individuals having CLBP including kinesiophobia, pain belief and fear avoidance belief, self-efficacy, anxiety, coping styles, sleep quality, locus of control and catastrophising. Social factors including occupation and demographic characteristics were also key in determining the quality of life. Pain and health-related factors such as pain severity and disability experienced by the patient, involvement in physical activity and health status have also contributed to the QOL in individuals with CLBP. The psychological status of the individual significantly contributed to the quality of life in the individuals’ having CLBP along with their physical status. Identifying the possible determinant is helpful in planning the rehabilitation strategies and intervention plans. The review identified some intervention strategies which are reported to manage the pain and improving the QOL including MBSR, Pilates methods and Back School programme. Due to the methodological limitations, any conclusions regarding the effectiveness of the identified interventions couldn’t be made. More exploration in the area could facilitate understanding regarding the interventions which are effective in improving the QOL in the context of CLBP.

## Data Availability

The data that support the findings of this study were derived from the databases (PubMed, ScienceDirect, PsyNet, and Google Scholar) available in the public domain.
